# Octopaminergic gene expression and flexible social behaviour in the subsocial burying beetle *Nicrophorus vespilloides*

**DOI:** 10.1111/imb.12090

**Published:** 2014-03-20

**Authors:** C B Cunningham, M K Douthit, A J Moore

**Affiliations:** 1 Department of Genetics, University of GeorgiaAthens, GA, USA

**Keywords:** aggression, mating strategies, octopamine, resource defence, social tolerance, tyramine, quantitative real-time PCR

## Abstract

Flexible behaviour allows organisms to respond appropriately to changing environmental and social conditions. In the subsocial beetle *Nicrophorus vespilloides*, females tolerate conspecifics when mating, become aggressive when defending resources, and return to social tolerance when transitioning to parenting. Given the association between octopamine and aggression in insects, we hypothesized that genes in the octopaminergic system would be differentially expressed across different social and reproductive contexts. To test this in *N. vespilloides*, we first obtained the sequences of orthologues of the synthetic enzymes and receptors of the octopaminergic system. We next compared relative gene expression from virgin females, mated females, mated females alone on a resource required for reproduction and mated females on a resource with a male. Expression varied for five receptor genes. The expression of *octopamine β receptor 1* and *octopamine β receptor 2* was relatively higher in mated females than in other social conditions. *Octopamine β receptor 3* was influenced by the presence or absence of a resource and less by social environment. *Octopamine α receptor* and *octopamine/tyramine receptor 1* gene expression was relatively lower in the mated females with a resource and a male. We suggest that in *N. vespilloides* the octopaminergic system is associated with the expression of resource defence, alternative mating tactics, social tolerance and indirect parental care.

## Introduction

The hallmark of adaptive behaviour is its flexibility, so that the appropriate behaviour is expressed at the appropriate time (West-Eberhard, 1989; Zayed & Robinson, 2012). For example, aggression towards a competitor is probably appropriate whereas aggression toward offspring or a mate is not. Such flexibility often leads to confusion and debates over nature and nurture, as variation in behaviour must reflect a genetic influence to evolve yet can change rapidly in response to current social and environmental conditions (Boake *et al*., 2002; Zayed & Robinson, 2012). It is increasingly apparent that some of this flexibility is associated with differential gene expression, especially when behavioural changes are rapid and reversible (Robinson *et al*., 2008; Bell & Robinson, 2011). Here, we investigate the possible changes in gene expression underlying behavioural flexibility in a subsocial beetle, *Nicrophorus vespilloides*, as it progresses through different adult life-history stages associated with reproduction. Changes in social behaviour are essential for successful reproduction in this species. To reproduce, an individual female must show flexibility in the timing of aggression, be able to express alternative mating tactics, develop social tolerance towards a mate and offspring, and switch to parental care at the appropriate time and place (Eggert & Müller, 1997; Scott, 1998).

*Nicrophorus vespilloides* has an unusual insect life history in that elaborate and extensive biparental or uniparental care by either a female or a male is required for successful reproduction (Eggert & Müller, 1997; Scott, 1998). After finding a vertebrate carcass, a potential parent buries the carcass, removes the external integument (hair, feathers, or scales) and forms the partially digested carcass into a ball. Throughout the period of parental care, the parent inhibits microbial growth on this resource by excreting antimicrobial solutions on the carcass (Scott, 1998) and removes fungus with its mouth; these behaviours are essential for successful reproduction and are considered ‘indirect’ parental care (Walling *et al*., 2008). The prepared carcass provides the sole food source for the developing offspring. For the first 24 h, *N. vespilloides* parents directly provision larvae with predigested carrion (direct care; Eggert & Müller, 1997; Scott, 1998; Walling *et al*., 2008). After 24 h, the offspring gradually transition to self-feeding (Smiseth *et al*., 2003) and disperse when the carcass is consumed. We hypothesize that the parental care of *N. vespilloides* involves the evolutionary elaboration and co-option of genes influencing three behavioural pathways: reproduction, mate and resource guarding, and food acquisition. This hypothesis derives from the prediction that parental care evolves in response to selection for the defence of offspring, to counter environmental adversity and to defend or supply essential resources to offspring, such as food (Tallamy, 1984; Tallamy & Wood, 1986; Clutton-Brock, 1991; Costa, 2006; Royle *et al*., 2012).

To begin elucidating the genetic controls underpinning behavioural flexibility in this beetle, we chose to characterize the octopaminergic system under several different social and reproductive contexts that vary greatly in their expectation for aggression, social tolerance and parental care. We chose the octopaminergic system because it is often involved in behaviours that require flexibility in their expression. The biogenic amine octopamine is an ancient and important control molecule that influences many aspects of arthropod life, including aggression and mating (Blenau & Baumann, 2001; Roeder, 2005; Verlinden *et al*., 2010a; Farooqui, 2012). It is synthesized through a two-step enzymatic process (tyrosine decarboxylase converts tyrosine to tyramine and tyramine β hydroxylase converts tyramine to octopamine) and exerts its influence through six G protein-coupled receptors belonging to three classes (Verlinden *et al*., 2010a; Farooqui, 2012). The three receptor classes, α, β (with three subtypes), and octopamine/tyramine (tyr; with two subtypes) are categorized by their affinities for octopamine and tyramine, intracellular signalling properties after activation and homology to vertebrate receptors (Verlinden *et al*., 2010a; Farooqui, 2012). Tyramine can also function as a neurotransmitter in addition to its role as a precursor molecule for octopamine synthesis (Lange, 2009).

We predicted that the octopaminergic system would be associated with the behavioural transition from aggression (defence) to social tolerance (mating, transition to parenting) and would respond to the presence or absence of a resource. We characterized both ligand and receptor components of this system given that both can influence behaviour. There is a well-established, taxon-wide positive association between octopamine and aggression (eg Adamo *et al*., 1995; Stevenson *et al*., 2005; Hoyer *et al*., 2008). Octopamine influences behavioural plasticity expressed through development, such as division of labour in honey bees (Schulz *et al*., 2002; Liang *et al*., 2012). Each class of octopaminergic receptors also influences more rapid behavioural flexibility. α and β receptors are associated with processes necessary for learning (Burke *et al*., 2012; Kim *et al*., 2013), changes in sociality (Verlinden *et al*., 2010b) and transitions to aggression (Rillich *et al*., 2011). β and tyr receptors are thought to play a role in olfaction and appetite, which suggests that their expression should change with the presence or absence of a resource (Kutsukake *et al*., 2000). Given the behavioural changes expressed by *N. vespilloides* as it transitions from mating to resource defence to parenting, we hypothesized that both the enzymes and receptors of the octopaminergic system would be differentially expressed under these different social and reproductive contexts.

To test our hypothesis that the octopaminergic system is involved in changes in *N. vespilloides* behaviour, we first identified eight orthologues of enzyme and receptor genes in this species: *tyrosine decarboxylase* (*tdc*), *tyramine β hydroxylase* (*tβh*), *octopamine β receptor 1* (*octβr1*), *octopamine β receptor 2* (*octβr2*), *octopamine β receptor 3* (*octβr3*), *octopamine α receptor* (*octαr*), *octopamine/tyramine receptor 1* (*tyrr1*) and *octopamine/tyramine receptor 2* (*tyrr2*). We next examined gene expression in four social/reproductive contexts: isolated, virgin females (providing baseline gene expression levels), mated females (social experience of mating), mated females given a reproductive resource (resource defence), and mated females given a reproductive resource and a male partner (reduced defence because of the presence of a social partner). These four contexts therefore provide the social and reproductive conditions under which we expect transitions to states where different appropriate behaviours can be expressed. We predicted that mating would not greatly influence the gene expression of the enzymes *tdc* or *tβh* as a single prior social experience should not change aggressiveness and there is no resource present. We predicted that mating might alter receptor gene expression because of octopamine's role in mating and reproduction. We predicted that genes involved with octopamine synthesis would be up-regulated when females were guarding a resource because of the need to express aggression in the context of resource defence. However, when females were paired with males on a resource, we predicted that the expression of octopamine synthesis enzyme genes would be lower to reflect increased social tolerance and the abdication of resource defence to the males. Simultaneously examining expression of all receptor genes allowed us to assess which responded, and differences in responses, to each of the social/reproductive contexts. This allows us to propose alternative hypotheses for gene function in this system.

## Results

### Sequence analysis of octopamine enzymes and receptors

**Figure 1:**
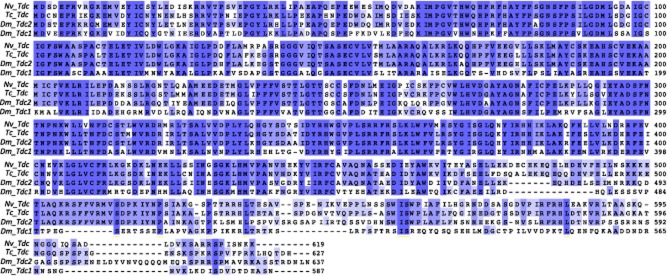
Boxshade of tyrosine decarboxylase (*Tdc*) proteins. Proteins were aligned with the ClustalW alignment algorithm on the Mobyle@Pasteur web portal with default settings and the boxshade was produced with JalView (v. 2.8). Shading is determined by the conservation of a residue at a position by percentage; dark blue = 100% of residues share identity, medium blue = 75% of residues share identity, light blue = 50% of residues share identity. The number at the end of each line of each protein sequence is the number of residues that a protein has up to the end of that line. Dm, *Drosophila melanogaster*; Nv, *Nicrophorus vespilloides*; Tc, *Tribolium castaneum*. GenBank accession numbers are provided in the Appendix.

**Figure 2:**
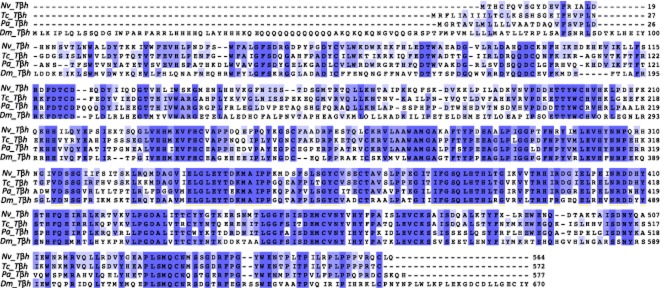
Boxshade of tyramine β hydroxylase (*tβh*) proteins. See Figure 1 legend for methods. Dm, *Drosophila melanogaster*; Nv, *Nicrophorus vespilloides*; Pa, *Periplaneta americana*; Tc, *Tribolium castaneum*.

**Figure 3:**
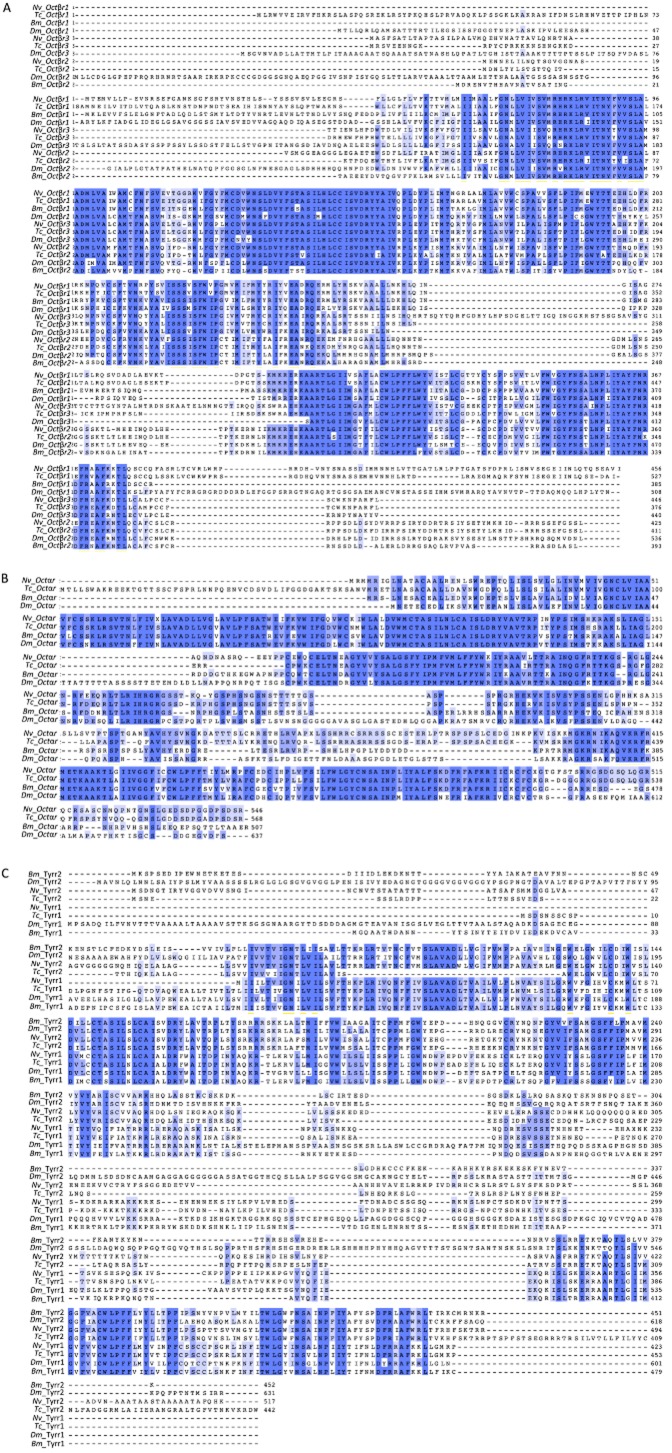
Boxshades of octopaminergic receptors. See Figure 1 legend for methods. Boxshade of (A) octopamine β receptors (*octβr*), (B) octopamine α receptors (*octαr*) and (C) octopamine/tyramine receptors (*tyrr*). Bm, *Bombyx mori*; Dm, *Drosophila melanogaster*; Nv, *Nicrophorus vespilloides*; Tc, *Tribolium castaneum*.

**Figure 4:**
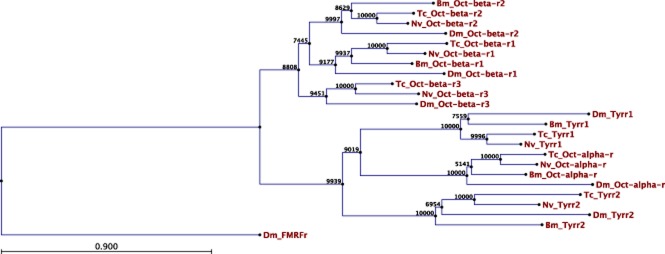
Phylogenetic tree showing the relationship of *Nicrophorus vespilloides* octopamine receptors to other known or putative octopamine receptors in insects. The tree was constructed with CLC Sequence Viewer software using the ClustalW alignment algorithm and a neighbour-joining tree construction method with 10 000 bootstraps. *Drosophila melanogaster* FMRFamide receptor was used as an outgroup to root the tree. Bm, *Bombyx mori*; Dm, *Drosophila melanogaster*; Nv, *N. vespilloides*; Tc, *Tribolium castaneum*. Scale bar represents substitution rate of amino acids per position. GenBank accession numbers are provided in the Appendix.

As octopamine itself has been associated with the control of behaviour (eg Adamo *et al*., 1995; Stevenson *et al*., 2005; Hoyer *et al*., 2008), we obtained full sequences for two enzymes involved in the synthesis of octopamine, *tdc* and *tβh*. The *tdc* sequence found was more similar to the neurally expressed *tdc* (*Dm*Tdc2) of *Drosophila melanogaster* than the peripherally expressed *tdc* (*Dm*Tdc1), 72 vs. 57% identity, respectively (Fig. 1). The *tβh* sequence showed high similarity to other functionally characterized *tβh*s (Fig. 2 ). We also identified full sequences for six of the expected octopamine receptors and follow the receptor nomenclature of Verlinden *et al*. (2010a). These sequences shared high similarity to other octopaminergic receptors. A boxshade analysis of all six receptors with representatives from multiple lineages showed a highly conserved portion of all of these receptors in the 3′ end of the proteins (Fig. 3). Phylogenetic analysis of the receptor sequences agreed with the assignments of identity based on BLAST searches of the National Center for Biotechnology Information (NCBI; Fig. 4). We have deposited all sequences in GenBank (accession numbers: *tdc-* KJ152556, *tβh*- KJ152557, *octβr1-* KJ152558, *octβr2-* KJ152559, *octβr3-* KJ152560, *octαr-* KJ152561*i tyrr1-* KJ152562, *tyrr2-* KJ152563).

### Gene expression

**Figure 5:**
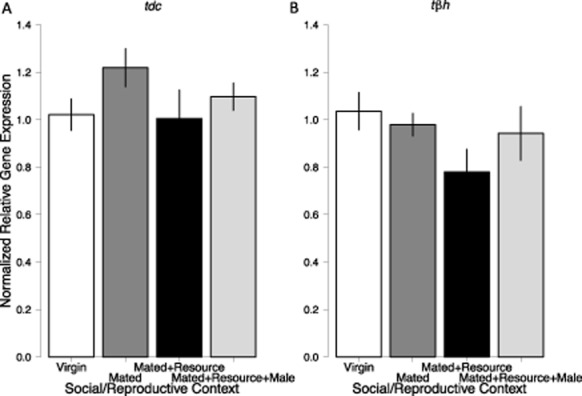
Normalized relative expression of enzyme genes in the octopaminergic system under different social and reproductive contexts. Bars are mean ± SEM (*N* = 10/treatment). Significance of each context compared with virgins was assessed using Dunnett's method. Neither (A) *tyrosine decarboxylase* (*tdc*) nor (B) *tyramine β hydroxylase* (*tβh*) were differentially expressed over the social and reproductive contexts assayed here.

We first examined gene expression of the enzymes involved with octopamine synthesis. Neither *tdc* (*F*_3,36_ = 1.325, *P* = 0.281) nor *tβh* (*F*_3,36_ = 1.584, *P* = 0.210) were differentially expressed across the different social and reproductive contexts (Fig. 5). This suggests that the synthesis of octopamine is not influenced by these social contexts.

**Figure 6:**
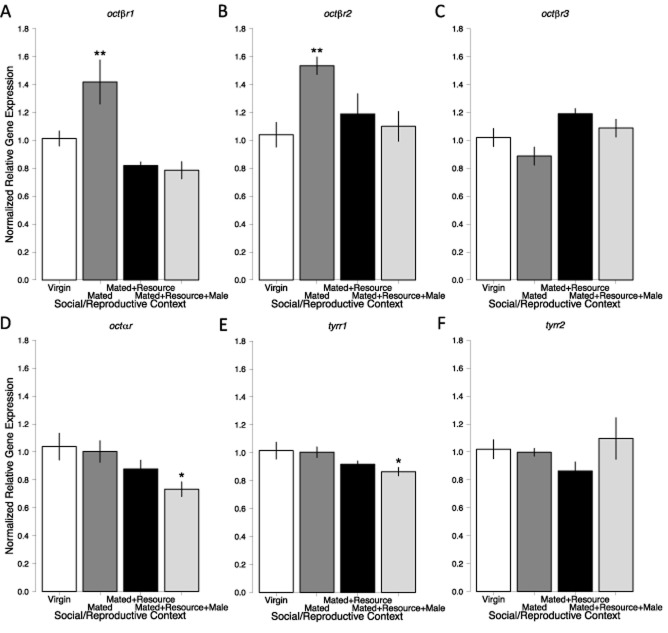
Normalized relative expression of octopaminergic system receptor genes under different social and reproductive contexts. Bars are mean ± SEM (*N* = 10/treatment). Significance of each context compared with virgins was assessed using Dunnett's method. Asterisks denote significance for *P* < 0.5 (*) and *P* < 0.01 (**). (A) Relative expression of *octopamine β receptor 1* (*octβr1*). (B) Relative expression of *octopamine β receptor 2* (*octβr2*). (C) Relative expression of *octopamine β receptor 3* (*octβr3*). (D) Relative expression of *octopamine α receptor* (*octαr*). (E) Relative expression of *octopamine/tyramine receptor 1* (*tyrr1*). (F) Relative expression of *octopamine/tyramine receptor 2* (*tyrr2*).

We next examined the gene expression of octopamine receptors (Fig. 6; Table 1). Overall, the social and reproductive contexts influenced most receptor gene expression levels, but there was not a consistent effect for any particular context. Compared to virgin females, there was significantly increased expression of octβr1 in mated females, but scientifically lower expression in solitary females with a resource and females with a resource and male (overall *F*_3,36_ = 10.434, *P* < 0.001; significant contrast − virgin vs. mated *P* = 0.008; Fig. 6A). The same pattern was seen in *octβr2* (overall *F*_3,36_ = 4.418, *P* = 0.01; significant contrast − virgin vs. mated *P* = 0.006; Fig. 6B). For *octβr3*, there was a significant change in expression in the different contexts, with expression increasing in the presence of a resource (overall *F*_3,36_ = 4.645, *P* = 0.008), but none of the specific pairwise *a priori* comparisons with virgins were statistically significant (Fig. 6C). Expression of *octαr* was significantly different across social contexts (overall *F*_3,36_ = 3.489, *P* = 0.025) with expression significantly lower when females were on a resource with a male compared to virgin females (*P* = 0.016; Fig. 6D). The same pattern was seen for *tyrr1*, with significant differences across social contexts (overall *F*_3,36_ = 3.18, *P* = 0.036) driven by significantly lower expression when on a resource with a male (*P* = 0.032; Fig. 6E). The expression levels of one receptor gene, *tyrr2*, was not significantly associated with the different social and reproductive contexts (overall *F*_3,36_ = 1.187, *P* = 0.328; Fig. 6F).

**Table 1:** Overall change in octopaminergic system gene expression in females under different social/reproductive contexts. Each cell indicates the *P* value comparing the expression level either across all treatments with an analysis of variance or that treatment compared with expression in the virgin treatment using Dunnett's method. Significant *P*-values followed by {+} indicate increased expression, those followed by {-} indicate decreased expression

	Gene	Overall model	Social/reproductive context		
Mated			Mated + Resource	Mated + Resource + Mate
Enzymes	*tdc*	*P* = 0.281	*P* = 0.249	*P* = 0.998	*P* = 0.862
	*tβh*	*P* = 0.210	*P* = 0.939	*P* = 0.113	*P* = 0.790
Receptors	*octβr1*	*P* < 0.001	*P* = 0.008 {+}	*P* = 0.306	*P* = 0.195
	*octβr2*	*P* = 0.010	*P* = 0.006 {+}	*P* = 0.628	*P* = 0.957
	*octβr3*	*P* = 0.008	*P* = 0.272	*P* = 0.122	*P* = 0.757
	*octαr*	*P* = 0.025	*P* = 0.973	*P* = 0.301	*P* = 0.016 {-}
	*tyrr1*	*P* = 0.036	*P* = 0.994	*P* = 0.221	*P* = 0.032 {-}
	*tyrr2*	*P* = 0.328	*P* = 0.996	*P* = 0.471	*P* = 0.871

*octαr*, *octopamine α receptor*; *octβr1−3*, *octopamine β receptor 1−3*; *tβh*, *tyramine β hydroxylase*; *tdc*, *tyrosine decarboxylase*; *tyrr1−2*, *octopamine/tyramine receptor 1−2.*

## Discussion

We had two main objectives with this study. First, we sought to identify the sequences of the genes in the octopaminergic system for the burying beetle, *N. vespilloides*. Second, we characterized expression of the two enzyme and six receptor orthologues under different social and reproductive contexts. We tested hypotheses about the role of the octopaminergic system in aggression, resource defence, social tolerance, and mating associated with the behavioural flexibility need for successful reproduction in this species. These behavioural traits are associated with gene expression in the octopaminergic system in various insects (Kutsukake *et al*., 2000; Verlinden *et al*., 2010b; Ishida & Ozaki, 2011; Koon *et al*., 2011; Burke *et al*., 2012; Liang *et al*., 2012; Zhou *et al*., 2012; Kim *et al*., 2013; Rein *et al*., 2013; Zhang *et al*., 2013) and are predicted to have been co-opted in the evolution of parental care (Tallamy, 1984; Tallamy & Wood, 1986; Clutton-Brock, 1991; Costa, 2006; Royle *et al*., 2012). Examining expression of these genes under controlled social conditions in *N. vespilloides*, a subsocial beetle that provides care to its young, therefore provides a novel test of the association between the octopaminergic system and several behaviours mediating successful social interactions.

Our experimental treatments were designed to reflect different social conditions that should have differing influences on the octopaminergic system as a female transitions through the stages associated with successful reproduction: mating, defence of resources, preparation of the resource, and providing parental care. First, we predicted that a brief mating experience alone represents minimal social interactions and would have little influence on the gene expression of the octopamine synthesis enzymes but might alter octopaminergic receptor gene expression. Second, we predicted that the enzymatic genes of the octopaminergic system would be up-regulated to promote resource defence or guarding (aggressive) behaviour when females were alone on a resource compared with virgins. Third, we predicted that the enzymatic genes of the octopaminergic system would be down-regulated to promote social tolerance between partners when females were on a resource with a male. Moreover, females are less likely to defend resources when males are present, which should also lower expression of enzymatic genes. Characterizing all of the receptors also allowed for potential specialization of these receptors to be assessed under these social and reproductive contexts.

Our results, although correlational, suggest a very individualized and subtle role for each octopaminergic gene in *N. vespilloides* across these social and reproductive contexts. We found changes in gene expression of multiple receptors, but not for genes transcribing the enzymes in the octopamine synthesis pathway. Patterns in gene expression were receptor specific, differing both in context and direction of change. Using virgin females as the *a priori* comparison treatment across the entire study, expression of two genes, *octβr1* and *octβr2*, was up-regulated by mating alone. One gene, *octβr3*, was up-regulated when a female was on a resource, both alone and with a male. Two genes, *octαr* and *tyrr1*, were down-regulated when a female was on a resource with a social partner. The diversity of responses suggests potential specialization of the receptors in *N. vespilloides*, such as that seen with *Drosophila* serotonin receptors (Johnson *et al*., 2009; Becnel *et al*., 2011).

There are several described roles for octopamine β receptors in a variety of behaviours and processes relevant to our social/reproductive contexts. Depression of Octβr's activity extends lifespan in male *Drosophila*, which suggests a role in resource allocation regulation (Spindler *et al*., 2013). *octβr2* has been linked to neural and behavioural plasticity, as well as memory reinforcement, in *Drosophila* (Koon *et al*., 2011; Burke *et al*., 2012). This receptor is also up-regulated in honey bees that seek novel food sources (Liang *et al*., 2012). Blocking β receptor activity does not depress aggression in variety of situations in crickets (Stevenson *et al*., 2005; Rillich *et al*., 2011; Rillich & Stevenson, 2011), suggesting a reduced role of these receptors in regulating aggression. Finally, an *octβr* in desert locust is also up-regulated when individuals transition from a solitary to social phase (Verlinden *et al*., 2010b). In our study, we found that *octβr1* and *octβr2* had increased expression in mated females, but not in females with a reproductive resource. A mated female without a resource is an ecologically relevant social condition for *N. vespilloides*, as females will mate even when there is no resource present (Eggert & Müller, 1997). The altered expression of *octβr1* and *octβr2* may reflect changes associated with adopting an alternative mating tactics associated with the lack of a resource required for breeding as female *N. vespilloides* off a resource are more choosey of mates and less tolerant of males (Beeler *et al*., 2002; House *et al*., 2007). It is also possible that these receptor genes are up-regulated to increase resource-seeking behaviour and then down-regulated once a resource is found. In *Drosophila*, *octβr3* has recently been shown to influence food-seeking behaviour (Zhang *et al*., 2013). Given that we found *octβr3* was more highly expressed whenever females were on a resource, this suggests there may be an association amongst the shift to indirect parental care, the expression of behaviours associated with preparation of food resources and the expression of this gene in *N. vespilloides*.

The octopamine α receptor also has several described roles. It influences behavioural changes through a role in memory formation, reinforcement and conditioning in *Drosophila* (Burke *et al*., 2012; Zhou *et al*., 2012; Kim *et al*., 2013). The expression of *octαr* also directly regulates neural activity to influence behavioural plasticity in honey bees (Rein *et al*., 2013). However, *octαr* was not differentially expressed when a solitary desert locust was grouped with other locusts (Verlinden *et al*., 2010b). In another nonsocial insect, blocking α receptor activity depressed aggression in crickets under several different contexts (Stevenson *et al*., 2005; Rillich *et al*., 2011). We found that *octαr* was expressed at significantly lower levels in females when they were on a resource with a male. In *Nicrophorus*, lone, mated females on a resource can reproduce but there is considerable competition for these resources and males that help defend the resource are tolerated (Müller *et al*., 2003). This suggests a potential association with *octar*, behavioural flexibility and a reduction of aggressive behaviour in *N. vespilloides* when a male is present to help defend the resource. Female *N. vespilloides* on a resource with a male are less likely to engage in aggressive encounters and are more often engaged in preparation of the carcass, a form of indirect parental care (Smiseth *et al*., 2005; Walling *et al*., 2008). They are more socially tolerant in general with a resource present. For example, females will accept and care for any larvae that arrive at the appropriate time (Müller & Eggert, 1990; Oldekop *et al*., 2007), accept males with little aggression (House *et al*., 2007; Trumbo, 2007) and recognize co-breeding males (Müller *et al*., 2003; Steiger *et al*., 2009). The potential for aggression and resource defence by females does exist, as they will readily and violently attack any intruding females (House *et al*., 2007; Hopwood *et al*., 2013).

Tyramine is a neurotransmitter with distinctive effects from octopamine, a role that has only recently been generally appreciated (Lange, 2009). *tyrr1* in *Drosophila* has been linked to olfaction (Kutsukake *et al*., 2000) and a tyramine receptor in blowflies has been suggested to influence appetite (Ishida & Ozaki, 2011). Blocking activity of tyramine receptors does not reduce aggression in crickets (Rillich *et al*., 2011; Rillich & Stevenson, 2011). Here, we found that *tyrr1* gene expression was down-regulated in females with a resource and male in *N. vespilloides*. It may be that females are eating less for themselves when preparing the resource, which would be consistent with the suggested role of appetite regulation in blowflies. However, females do feed from the resource although it is not clear if they reduce their overall food intake. Carcass preparation, however, is a part of the indirect parental care that females provide to offspring (Walling *et al*., 2008). Overall, our results suggest that expression of *tyrr1* and *octar* are associated with the transition to parental care.

The octopaminergic system reflects behavioural changes associated with different social conditions in the burying beetle *N. vespilloides*. Although the associations are not as simple as we predicted, associated with more than just the propensity for aggression, multiple different associations with behavioural changes is consistent with the known roles for the different octopamine receptors in a variety of insects. In particular, the octopaminergic system in *N. vespilloides* appears to be associated with resource defence, alternative mating tactics and transitions to social tolerance and parenting. A more fine-scale study looking at specific subpopulations of octopaminergic neurones within the brain might help to refine some potential functions of the differentially expressed receptors as the same biogenic amine receptor can have different expression profiles within different neuronal subpopulations even within the same anatomical brain region (McQuillan *et al*., 2012), which is variation that our study did not capture. To test the specific hypothesized roles for the octopaminergic system in *N. vespilloides* will require the demonstration of a causal association and thus the development of additional genetic tools and manipulations. It may be that there is specialization of octopamine receptors associated with or facilitating the evolution of subsociality.

## Experimental procedures

### Insect colony and husbandry

We obtained an outbred colony of *N. vespilloides* founded from a recently derived, outbred population maintained at the University of Exeter, Cornwall, UK (Head *et al*., 2012). The beetles were kept in a common, constant temperature room set at 22 ± 1 °C, under a 15:9 light : dark cycle, and fed decapitated mealworms (*Tenebrio* sp.) *ad libitum* once a week after adult eclosion. We housed beetles individually at dispersal in 9-mm-diameter and 4-mm-deep circular biodegradable plastic deli containers (Eco products, Boulder, CO, USA) filled with 2.5 cm of moist soil (Pure Organic Potting mix; Jungle Growth LLC, Statham, GA, USA).

### Sequence analysis

We extracted total RNA from whole heads of virgin *N. vespilloides* collected into liquid nitrogen using a Qiagen RNeasy Lipid mini-kit (Qiagen, Valencia, CA, USA). Tissues were first powdered in liquid nitrogen with a mortar and pestle followed by hand-held motorized pestle homogenization (Kimble Chase, Vineland, NJ USA) after submersion into the QIAzol lysis buffer. We quantified RNA with a Qubit 2.0 fluorometer (Invitrogen Corporation, Carlsbad, CA, USA) according to the manufacturer's instructions. We synthesized cDNA with Quanta Biosciences qScript reverse transcriptase master mix (Quanta Biosciences, Gaithersburg, MD, USA) following the manufacturer's instructions from 500 ng total RNA. RNA was stored at −80 °C and cDNA was stored at −20 °C.

We identified putative genes belonging to the octopaminergic system by interrogating an unpublished draft genome and three separate unpublished transcriptomes with known or predicted proteins sequences of the genes of interest (GOIs) from *Drosophila* and *Tribolium* using the tBLASTn (v2.2.25+) search algorithm (Altschul *et al*., 1997). Briefly, the draft genome was assembled from a next-generation sequencing (NGS) data set from a single inbred larva. The three transcriptomes were assembled from NGS data sets following standard RNA-Seq and ChIP-Seq protocols assessing differences across behavioural states in a breeding cycle (virgin, mated, caring, postcaring). *tdc*, *tβh*, *octβr1*, *octβr2*, *octβr3*, *octαr*, *tyrr1* and *tyrr2*) all had putative candidate loci identified. This process was also carried out for several widely used endogenous control genes: elongation factor 1α (*ef1α*), glyceraldehyde 3-phosphate dehydrogenase (*gapdh*) and tata-box binding protein (*tbp*).

We ran 50 μl PCR reactions using Phusion polymerase (Thermo Scientific, Pittsburgh, PA, USA) according to the manufacturer's recommendation with the addition of 1.5 μl dimethyl sulphoxide and a target of 100 ng of cDNA template per reaction. We used a touchdown PCR temperature profile with an initial 5-min 95 °C denaturation step followed by eight cycles of denaturation at 95 °C for 30 s, an annealing step that descended by 1 °C each cycle starting at 63 °C, and an elongation time of 60 s per 1 kb of a target amplicon at 72 °C. This was followed by 35 cycles of amplification with the same settings except that the annealing temperature was kept constant at 55 °C. We separated PCR products on 1% agarose gels and stained with ethidium bromide. We purified correctly sized products with a Qiagen QIAquick PCR purification protocol or with a QIAquick gel purification protocol after size selection if multiple bands were present following the manufacturer's instructions. Purified products were sequenced with a Sanger capillary sequencing protocol. We assembled individual sequencing runs with Sequencher (v. 5.01, http://genecodes.com) using default settings. We then compared our sequences against NCBI's nonredundant protein database (http://blast.ncbi.nlm.nih.gov) for all insects to determine identity using the BLASTx algorithm.

If putative GOI sequences were incomplete, we used the consensus PCR-validated sequences to re-interrogate the four genomic resources available for *N. vespilloides* with the BLASTn (v. 2.2.25+) algorithm (Zhang *et al*., 2000). From the collection of contigs and PCR sequences, we assembled putative full sequences for all eight GOIs. To visualize high conservation portions of the proteins across multiple lineages, we aligned the predicted *Tribolium castaneum* sequence of each protein with the functionally characterized sequences from *D. melanogaster*. We obtained raw alignment files of the proteins with the ClustalW algorithm with the Mobyle@Pasteur web portal (http://mobyle.pasteur.fr) and imported these into JalView (v. 2.8) (Waterhouse *et al*., 2009) to produce boxshades.

To further establish the identity of the putative octopaminergic system receptor genes of *N. vespilloides*, we constructed a phylogenetic tree with our translated putative gene sequences and known or predicted receptor protein sequences (Verlinden *et al*., 2010a). We aligned sequences with the ClustalW algorithm and constructed the tree with the neighbour-joining method as implemented in CLC Sequence Viewer (v. 6.8.2, http://www.clcbio.com) using the default settings with 10 000 bootstraps to estimate the stability of the relationships.

### Gene expression analysis

To test the hypothesis that octopaminergic gene expression is influenced by social or reproductive context, we created four treatment groups that were all harvested at 13 days post-adult eclosion. *a priori*, we chose virgin females (*Virgin*) as the treatment that we would compare to the other social/reproductive treatments. This treatment held individual beetles in isolated containers from larval dispersal until tissue collection. These animals had no social encounters, no ability to reproduce and no opportunity to reproduce. Our second treatment was mated females (*Mated*). These females had a single mating encounter with a male when they were 10 days post-adult eclosion in a mating box (17.2 × 12.7 × 6.4 cm; Pioneer Plastics, Dixon, KY, USA) filled with ∼1 cm of soil. The males were 10–14 days post-adult eclosion and the encounter lasted 4 h, which is more than sufficient time for a female to obtain a lifetime supply of sperm (House *et al*., 2008). We returned both the females and males to their original containers after this 4-h period until tissue collection. This treatment therefore represents a relatively brief social encounter, providing the ability, but not opportunity, to reproduce. With the third treatment, mated females on a reproductive resource without a male (*Mated* + *Mouse*), we gave females the resource required to reproduce but insufficient time for larvae to hatch. We treated the females as in the *Mated* treatment, but 24 h following mating we placed the females in a new mating container half filled with moist soil and with a 20–24 g mouse carcass present. Thus, these animals had a brief social encounter and an opportunity to reproduce under uniparental conditions. Finally, the fourth treatment consisted of a mated female on a reproductive resource with a male (*Mated* + *Mouse* + *Male*). Again, we treated the females as in the *Mated* treatment. The following day, we placed the same female−male mating pair into a new mating container half filled with moist soil and with a 20–24 g mouse carcass present. Thus, these animals had a brief social encounter followed by an extended social encounter, and an opportunity to reproduce under biparental conditions. There were 10 replicates of each treatment.

We collected females at 13 days post-adult eclosion regardless of treatment and dissected out brains submerged in ice-cold 1× phosphate-buffered saline (National Diagnostics, Atlanta, GA, USA). We submerged samples into 100 μl RNAlater (Ambion, Grand Island, NY, USA) on ice and then followed the manufacturer's protocol for storage at −20 °C until RNA extraction.

We extracted total RNA from single dissected brains using a Qiagen RNeasy micro kit following the manufacturer's instructions with the addition of 700 μl QIAzol (Qiagen) as the lysis buffer and 150 μl chloroform (J.T. Baker, Center Valley, PA, USA) after homogenization. We also treated samples with DNase I (Qiagen) on column according to the manufacturer's instructions to help ensure minimal genomic DNA contamination. We quantified RNA with a Qubit 2.0 fluorometer according to the manufacturer's instructions. We synthesized cDNA with Quanta Biosciences qScript reverse transcriptase master mix following the manufacturer's instructions from 500 ng total RNA. We generated multiple no-template controls for each social/reproductive context following the same protocol as experimental samples, except that RNAse-free water was substituted for qScript master mix during the cDNA synthesis step. RNA was stored at −80 °C and cDNA was stored at −20 °C.

We designed quantitative real-time PCR (qRT-PCR) primers from the PCR-validated consensus sequences of each of our GOIs and several endogenous control genes (*ef1α*, *gapdh* and *tbp*) using Primer3 (v. 4.0.0; Untergrasser *et al*., 2012). Multiple primer pairs (18-23mers) for each gene were designed to produce similarly sized amplicons (90–110 bp) and to flank exon boundaries using the draft genome of *N. vespilloides* as a reference. Primer pairs were then validated by estimating PCR efficiency and the number of amplicons generated from each pair was assessed with a disassociation curve from a qRT-PCR run. PCR efficiency was estimated with a four-point, four-fold serial dilution series using a pool of common cDNA, which had been generated using the same protocol as the experimental samples. This dilution series produced a linear dynamic range encompassing the experimental variation in threshold cycle (*C*_T_) values of all target amplicons. It also ensured that primer pairs with efficiencies close to two were used to meet the assumptions of the ΔΔ*C*_T_ method. To further ensure that a single amplicon per primer pair was produced, we separated one qRT-PCR reaction on a 1% agarose gel stained with ethidium bromide.

We ran qRT-PCR with Roche LightCycler 480 SYBR I Green Master Mix using a Roche LightCycler 480 (Roche Applied Science, Indianapolis, IN, USA). We ran triplicate technical replicates (*N* = 10 of each treatment) using 10 μl reactions containing 5 μl SYBR mix, 2 μl cDNA diluted 1:10 with qRT-PCR grade water, and 3 μl of a primer stock containing both the forward and reverse primers at 1.33 μmol/l. We set the temperature profile according to the manufacturer's instructions for an enzyme activation step, followed by 45 cycles of amplification at 60 °C and a dissociation curve step to assess the number of amplicons generated with each reaction.

To establish the stability of endogenous control genes, we ran replicates of samples from the different social/reproductive contexts while controlling for input cDNA amount. Single-strand cDNA was quantified with a Qubit 2.0 fluorometer according to the manufacturer's instructions after treating samples with RNaseH. cDNA was diluted so that each technical replicate contained 1.5 ng cDNA. We assessed the stability of endogenous control gene amplicons by visual inspection of *C*_T_ values and found that *tbp* did not vary across social contexts. Even when aliquoting cDNA from a diluted pool rather than aliquoting a standard amount of cDNA, there was no statistically significant difference in *tbp* expression across our social and reproductive contexts (*F*_3,36_ = 0.286, *P* = 0.836). On the experimental qRT-PCR plates, we ran multiple no-template controls. Additional information suggested by the Minimum Information for Publication of Quantitative Real-Time PCR Experiments (MIQE) guidelines can be found in the Appendix.

We used the ΔΔ*C*_T_ method (Livak & Schmittgen, 2001) to convert raw expression data to normalized relative expression values, using the virgin treatment as our comparison group. All values were normally distributed. Data were visually inspected for outliers. We tested for the effect of social/reproductive context using an analysis of variance, followed by Dunnett's method (Dunnett, 1955) for multiple *a priori* comparisons using virgins as the control group. We used JMP Pro (v. 10.0.1, http://jmp.com) for all statistical analyses.

## Appendix

### Additional information about the quantitative real-time PCR protocol and Figs 4

- Additional information suggested in the Minimum Information for Publication of Quantitative Real-Time PCR Experiments (MIQE) guidelines not already provided in paper.
  - Quantitative real-time PCR (qRT-PCR) primer sequences
    *tdc*-
      forward: GTCATACGGTTTTGCGCTGT
      reverse: AATAATTCGCTGGCATATTCTGTT
      *tβh*-
      1. forward: CAGAGATGGCATCGAGTTA
      2. reverse: GCATCACCCGGTAGAACTTT
      *octβr1*-
      1. forward: GGCATCATCGTGTCCGCTTT
      2. reverse: ACGGATGGTGGACTGTAGCA
      *octβr2*-
      1. forward: TTCGCCATGACCTTCAA
      2. reverse: CGAATTCCAGACGTCGCACA
      *octβr3*-
      1. forward: CAACACCGCCCTAAACACGA
      2. reverse: CCCTCCAGCTCTTCGACTGT
      *octαr*-
      1. forward: CGCAGGTCAAACGCTTCAGA
      2. reverse: GGTGAAGAAGGGTAGCCAGC
      *tyrr1*-
      1. forward: CGGATCCCATAAACTACGCGC
      2. reverse: GGCCAATCGTTCCAACCGAT
      *tyrr2*-
      1. forward: GTGTGGATAAGTTCGGCGCT
      2. reverse: CGTAGCCCGTGTTCTTGTTGT
      *tbp*:
      1. forward: CACCCATGACTCCAGCAGAT
      2. reverse: ACGTGCATGCAGAGCTATCTT
    Primers were manufactured by Integrated DNA Technology (IDT, Coralvill, IA, USA) and purified with IDT's standard desalting technique.
    - qRT-PCR validation
      Primer efficiency
      *tdc*: 2.01 (*r*^2^_calibration curve _=_ _0.993)
      *tβh*: 2.10 (*r*^2^_calibration curve _=_ _0.993)
      *octβr1:* 1.98 (*r*^2^_calibration curve _=_ _0.990)
      *octβr2:* 1.96 (*r*^2^_calibration curve _=_ _0.997)
      *octβr3:* 1.93 (*r*^2^_calibration curve _=_ _0.965)
      *octαr:* 1.97 (*r*^2^_calibration curve _=_ _0.994)
      *tyrr1:* 1.94 (*r*^2^_calibration curve _=_ _0.976)
      *tyrr2*: 1.96 (*r*^2^_calibration curve _=_ _0.996)
      *tbp*: 2.16 (*r*^2^_calibration curve _=_ _0.989)
    - Data analysis
      No Template Control samples
      9/10 samples had no amplification; 1/10 samples had inconsistent amplification >5 cycles after experimental values.
  - GenBank accession numbers for non- *Nicrophorus vespilloides* protein sequences used in the boxshade and phylogenetic tree figures
    Figure 1. Tyrosine decarboxylase
    *Tribolium castaneum*: EFA10348.1
    *Drosophila melanogaster*: Tdc1-A1Z6N2, Tdc2-A1Z6N4
    Figure 2. Tyramine-β-hydroxylase
    *Tribolium castaneum*: XP_974169.1
    *Drosophila melanogaster*: Q86B61
    *Periplaneta americana*: I7CTE1
    Figures 3 and 4. Octopaminergic receptors
    If available, sequence from Verlinden *et al*. (2010a) were used.
    Octβr1
    *Tribolium castaneum*: XP_974265.1
    *Drosophila melanogaster*: Q9VCZ3
    *Bombyx mori*: XP_004922133.1
    Octβr2
    *Tribolium castaneum*: XP_974214
    *Drosophila melanogaster*: Q4LBB9
    *Bombyx mori*: NP_001171666.1
    Octβr3
    *Tribolium castaneum*: XP_974238
    *Drosophila melanogaster*: Q4LBB6.4
    Octαr
    *Tribolium castaneum*: EFA10678
    *Drosophila melanogaster*: ACC17442
    *Bombyx mori*: NP_001091748.1
    Tyrr1
    *Tribolium castaneum*: NP_001164311.1
    *Drosophila melanogaster*: AAA28731
    *Bombyx mori*: CAA64865
    Tyrr2
    *Tribolium castaneum*: EFA_10716.1
    *Drosophila melanogaster*: NM_142844
    *Bombyx mori*: BAI52937
    FMRFamide Receptor (used only in phylogenetic tree)
    *Drosophila melanogaster*: NP_647758
